# *HAAO* rs3816183 Polymorphisms [T] Increase Anterior/Middle Hypospadias Risk in Southern Han Chinese Population

**DOI:** 10.3389/fped.2022.842519

**Published:** 2022-03-21

**Authors:** Yanqing Liu, Wen Fu, Kai Fu, Xiaoyu Zuo, Wei Jia, Ning Wang, Yan Zhang, Guochang Liu, Fuming Deng

**Affiliations:** ^1^Guangzhou Women and Children's Medical Center, Guangzhou Medical University, Guangzhou, China; ^2^Department of Urology, Guangzhou Women and Children's Medical Center, Guangzhou Medical University, Guangzhou, China; ^3^Guangdong Provincial Key Laboratory of Research in Structural Birth Defect Disease, Department of Pediatric Surgery, Guangzhou Women and Children's Medical Center, Guangzhou Institute of Pediatrics, Guangzhou Medical University, Guangzhou, China

**Keywords:** hypospadias, *HAAO*, single-nucleotide polymorphism (SNP), genetics, urethral abnormalities

## Abstract

Hypospadias is one of the most common congenital external genital malformations, which is characterized by abnormal urethral meatus. However, the etiology remains to be incompletely understood. *HAAO* is a gene that encodes a protein, which catalyzes the synthesis of quinolinic acid, and has been identified as a risk gene for hypospadias. Thus, this study was conducted to elaborate the association between *HAAO* gene polymorphism rs3816183 T>C and hypospadias in the largest hypospadias cohort from Asia, including 577 patients and 654 healthy controls in China. The strength of interrelation was evaluated using 95% confidence intervals (CIs) and odds ratios (ORs). Based on the stratified analysis of hypospadias subtypes, it was found that the *HAAO* risk allele rs386183[T] enhances the susceptibility for hypospadias among patients with anterior/middle hypospadias subtypes (adjusted OR = 1.31, 95% CI = 1.05–1.64, *p* = 0.017). Enhanced risk of hypospadias in the entirety could not be demonstrated (OR = 1.20, 95% CI = 1.00–1.47, *p* = 0.054). In summary, our study found that the rs3816183[T] polymorphism is associated with increased risk of anterior/middle hypospadias among Southern Han Chinese children. The mechanisms by which the variations in the *HAAO* gene require further research.

## Background

Hypospadias is one of the most common congenital external genital malformations, which is characterized by abnormal urethral meatus (1), and affects approximately 20.9 out of every 10,000 births and has shown significant increases worldwide (2). Over the past decade, an increasing trend in the prevalence of hypospadias has been observed in China (3, 4). The clinical characteristics of hypospadias include proximal urethral opening, ventrally deficient hooded prepuce, and chordee (5). Hypospadias can be classified into two subgroups based on the urethral meatus location: anterior/middle hypospadias and posterior hypospadias (6). The meatus localization is best evaluated during surgery when chordee is corrected.

Although the surgical approach to hypospadias treatment has a great progress over the past decades, its etiology remains incompletely understood (1, 7–9). Individual phenotypic differences, such as disease susceptibility, survival, and treatment response, were identified to be associated with different genetic variants (10). Genetic variants have been observed to be associated with hypospadias risk (11, 12). However, very few studies have focused on variants in potential genes, such as *DGKK, MAMLD1, MID1, CYP1A1, GSTM1*, and *GSTT1*, which are associated with susceptibility to hypospadias (9). Some single-nucleotide polymorphisms (SNPs) have been reported in association to hypospadias. Nevertheless, recent studies used small sample sizes and have not been consistently replicated (13, 14).

Geller et al. conducted a genome-wide association study (GWAS) and reported that 17 SNPs were independently associated with hypospadias (15). Yoshiyuki validated these 17 SNPs in a Japanese cohort. However, only *HAAO* rs3816183 T>C was significantly associated with an increased risk toward hypospadias (16). Considering that ethnic differences exist at some loci, it would prove meaningful to evaluate the effect of SNPs on hypospadias susceptibility in different ethnic groups. Thus, we conducted this study to validate the association of *HAAO* rs3816183 T>C polymorphism with hypospadias susceptibility.

## Materials and Methods

### Study Population

We recruited 557 isolated hypospadias patients at the Guangzhou Women and Children's Medical Center from January 2016 to December 2019, all of whom were Han Chinese, and the diagnosis was confirmed by pediatric urologists before surgery repair. Hypospadias classification was performed by experienced pediatric urologists at our center. The meatus localization is best evaluated during surgery when chordee is corrected. Based on the urethral orifice, the patients were divided into two groups: patients with anterior/middle hypospadias were defined as having a urethral opening in glanular, subcoronal, distal penile, and midshaft penile areas, while patients with posterior hypospadias were identified as having the urethral opening in penoscrotal, scrotal, and perineal areas. The control group included 654 male children without a medical history of hypospadias, who were selected from the Guangzhou Women and Children's Medical Center. Since hypospadias can be inherited, all the patients and controls group with a first-degree relative who suffers from hypospadias were excluded.

Informed consent was obtained from all patients' parents or legal guardians. This study was approved by the Ethics Committee of Guangzhou Women and Children's Medical Center in China.

### DNA Extraction and Genotyping

Genomic DNA was extracted from venous blood samples using TIANamp Blood DNA kits (Catalog No. DP335-02; TIANGEN Biotech Co. Ltd., Beijing, China) following the manufacturer's instructions (17). NanoPhotometer® N50 (Implen GmbH., Munich, Germany) was used to assess DNA purity and concentration. Genomic DNA was amplified using the ABI-7900 real-time quantitative PCR instrument (Applied Biosystems, Foster City, CA, USA) and was subjected to *HAAO* rs3816183 TaqMan genotyping (18). PCR reactions were run as described in the previous study (19) using TaqMan® SNP Genotyping Assays (Catalog No: 4351379,C_180222_20, Thermo Fisher, USA) and TIANexact genotyping qPCR PreMix (Probe) (Catalog No. FP211-02; TIANGEN Biotech Co. Ltd., Beijing, China). In addition, 10% of DNA samples were selected randomly for second genotyping. The accuracy of data was ensured by the replicated samples with 100% consistency (19).

### Statistical Analysis

SAS 9.4 software (SAS Institute Inc., Cary, NC, USA) and GraphPad Prism version 8 (GraphPad Software, Inc., La Jolla, California, USA) were used to perform statistical analyses. Hardy–Weinberg equilibrium (HWE) test was performed in the control group using a goodness-of-fit chi-squared test. SNPs were analyzed for association with hypospadias susceptibility by comparing the risk of allele frequency (allelic test) in patients and controls, along with other tests using PLINK 1.9 (20). Association was stratified by subgroup through comparing controls with cases with a certain subgroup. A *p*-value of 0.05 was considered statistically significant (21).

## Results

### Association Between *HAAO* rs3816183 Polymorphism and Hypospadias Susceptibility

In the present study, 534 of 557 patients and 634 of 654 controls could be successfully genotyped. The frequencies of controls and patients group genotypes are shown in Table 1. The frequency distribution of the rs3816183[T] genotype in the control groups was consistent with HWE (*p* = 0.64). The HAAO rs3816183 TT phenotype was associated with an increased risk of hypospadias (TT vs. CC: OR = 1.57, 95% CI = 1.12–2.19, *p* = 0.008). Nevertheless, the results showed that the *HAAO* rs3816183[T] polymorphism may not be associated with hypospadias susceptibility in dominant and recessive models (adjusted OR = 1.19, *p* = 0.15/adjusted OR = 1.59, *p* = 0.06).

**Table 1:** Association between HAAO rs3816183 T>C polymorphism and hypospadias susceptibility.

**Genotype**	**Cases** **(*n* = 557)**	**Controls** **(*n* = 654)**	**Crude OR (95% CI)**	***p***	**Adjusted OR (95% CI)1**	***p* ^*a*^**
CC	288	376	1.0			
TC	204	232	1.09 (0.85–1.39)	0.52	1.09 (0.87–1.39)	0.52
TT	42	35	1.57 (1.12–2.19)	**0.008**	1.57 (1.12–2.19)	**0.008**
Genotypic				0.13		0.11
Dominant (TT+TC vs. CC)	246/288	267/376	1.20 (0.95–1.52)	0.12	1.19 (0.94–1.52)	0.15
Recessive (TT vs. CC+TC)	42/492	35/608	1.48 (0.93–2.36)	0.10	1.59 (0.99–2.57)	0.06

*Values are shown as numbers. Significant p values (<0.05) are in bold. CC, homozygous protective; TC, heterozygous; TT, homozygous risk for rs3816183; OR (95% CI), odds ratio and confidence interval. ^a^Adjusted for age*.

### Stratification Analysis of *HAAO* Gene Polymorphism With Hypospadias Susceptibility

Hypospadias can be divided into different subtypes based on the urethral meatus location after penile degloving. The *HAAO* risk allele rs3816183[T] was associated with an increased susceptibility toward anterior/middle hypospadias (OR = 1.35, 95% CI = 1.08–1.68, *p* < 0.01). Nevertheless, no significant association was found between the *HAAO* risk allele rs3816183 T and patients with posterior hypospadias (OR = 1.03, 95% CI = 0.80–1.32, *p* = 0.81).

## Discussion

Hypospadias is a complex, congenital, external genitalia malformation. Genetic factors are important causative reason in the development of hypospadias (11, 12). Kojima et al. replicated rs3816183 of *HAAO* polymorphism with hypospadias and found that rs3816183 [T] was significantly increased the hypospadias susceptibility toward both posterior and anterior/middle hypospadias (16). However, the *HAAO* rs3816183 polymorphism was only significantly associated with an increased susceptibility toward anterior/middle hypospadias susceptibility in the present study. Therefore, our study demonstrated that *HAAO* rs3816183 polymorphism is not equally associated with hypospadias risk in different populations.

**Figure 1:**
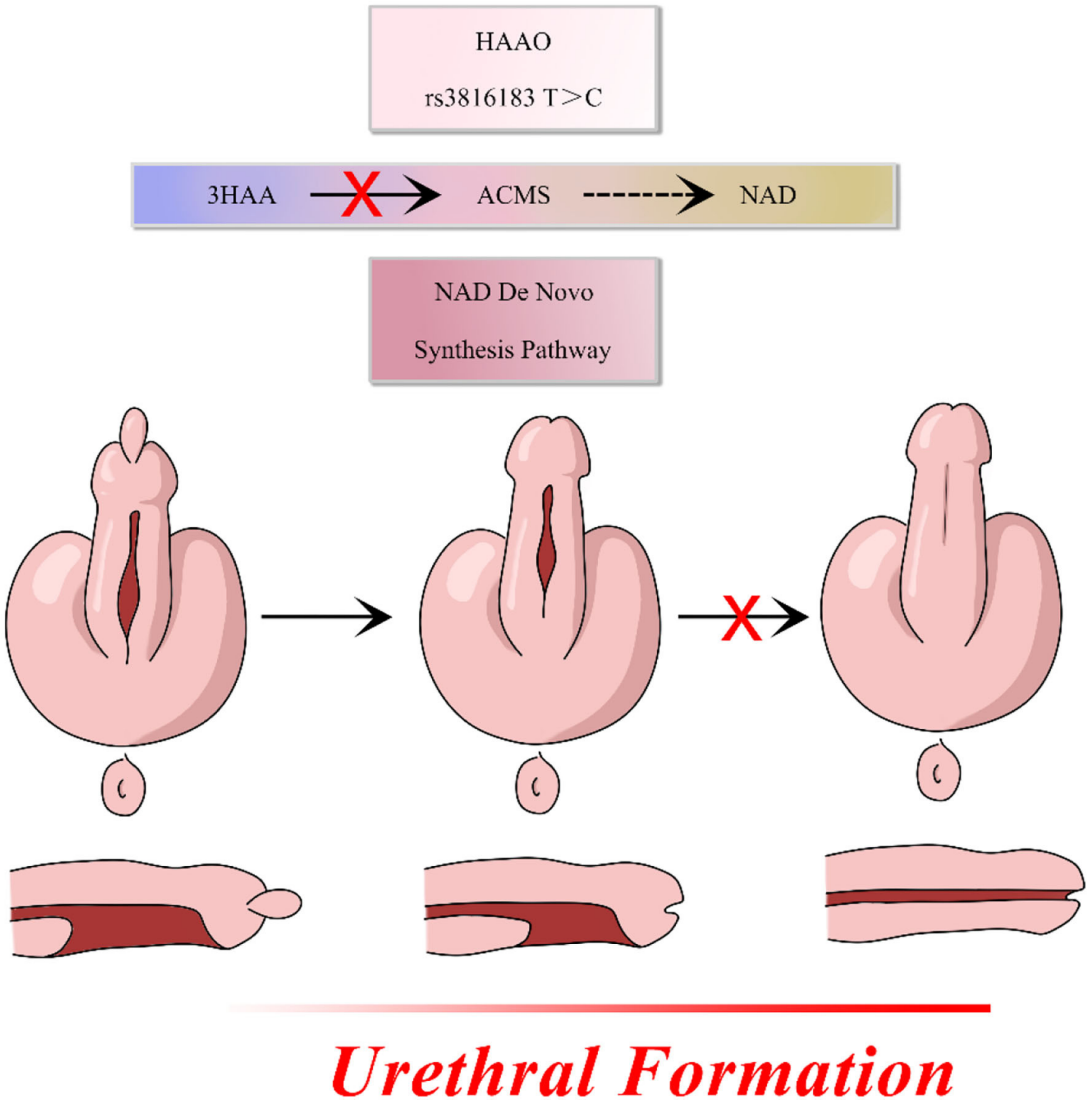
HAAO rs3816183 T>C polymorphism may disrupt the metabolism of its encoded protein leading to disorders of NAD synthesis, which contribute to the pathogenesis of hypospadias.

The *HAAO* gene, which is widely distributed in various organs (22–24), encodes a protein that catalyzes the synthesis of quinolinic acid (QUIN) from 3-hydroxyanthranilic acid. Huang et al. showed that hypermethylation of the HAAO gene predicts disease-free survival in patients with endometrioid endometrial cancer (25). Martin et al. reported that hypercholesterolemia and atherosclerosis may be treated and prevented by targeting the *HAAO* gene (26). Previous studies have demonstrated that the *HAAO* gene is associated with cancer biomarkers and degenerative diseases. The relationship between the *HAAO* gene and developmental disorders has also been reported. *HAAO* has also been correlated with congenital malformations and miscarriage and, when combined with environmental factors, may impair embryo outcomes (27). Pathogenesis of hypospadias has been attributed to the incomplete fusion of the urethra in a portion of the penis and the expression of *HAAO* in male mouse genital tubercle. Moreover, genetic variants of *HAAO* may specifically impede the migration and proliferation of normal urethral cells. We hypothesized that the *HAAO* rs3816183 T>C polymorphism may disrupt the metabolism of its encoded protein leading to disorders of NAD synthesis, which contribute to the pathogenesis of hypospadias (Figure 1). Similar genetic studies have suggested that rs3816183[T] *HAAO* polymorphisms may result in increased hypospadias susceptibility (16). However, in our study, the association between rs3816183 T>C *HAAO* polymorphism and hypospadias susceptibility was observed in anterior/middle group but not in posterior hypospadias patients. This discrepancy could be attributed to the sample size and ethnic differences in patients. In addition, causes of hypospadias may be genetic, maternal, environmental, or a combination of all of these factors. Posterior hypospadias have been reported to be associated with maternal factors, such as oligohydramnios, premature birth, and hypertension, suggesting that the underlying placental insufficiency may be an important contributing factor (28). Environmental factors, such as phthalates, have been associated with a toxic effect on the male reproductive system and the development of hypospadias (29). The fact that there may be many complex causes for hypospadias and that the environmental and maternal factors were not accounted for in our study could be the reason that the *HAAO* rs3816183 variants was found to be associated only with anterior/middle hypospadias.

This is the largest Asian case–control study to investigate the association of *HAAO* polymorphism rs3816183 T>C with hypospadias susceptibility. Our results demonstrated that the SNPs rs3816183[T] in *HAAO* may be associated with increased anterior/middle hypospadias but not posterior hypospadias (Table 2), suggesting that *HAAO* may influence distal part of penile urethral formation.

**Table 2:** Stratification analysis to evaluate the association between *HAAO* rs3816183 T>C polymorphism and hypospadias susceptibility (by subgroup).

**rs3816183**	**A1**	**AF of cases**		**AF of controls**	**Cases vs. controls**		**Posterior vs. controls**		**Anterior/middle vs. controls**	
		**Posterior**	**Anterior/middle**		***p***	***OR (CI95)***	***p***	***OR (CI95)***	***p***	**OR (CI95)**
	T	0.24	0.29	0.23	0.054	1.20 (1.00–1.47)	0.81	1.03 (0.80–1.32)	**0.017**	1.31 (1.05–1.64)

*A1, effect allele; AF, allele frequency of effect allele. Significant p values (<0.05) are in bold*.

However, there were some limitations to this study. First, environmental factors, such as difference in diet and geographic locations, were not analyzed. Second, in-depth exploration of *HAAO* rs3816183T>C and hypospadias sensitivity mechanisms is required. This may have potential implications for hypospadias prevention. Finally, multiple center studies are warranted to confirm our findings.

## Conclusion

The *HAAO* rs3816183[T] is associated with increased risk to anterior/middle hypospadias in Southern Han Chinese population. Our findings support the hypothesis that the mechanism underlying the variations in the *HAAO* gene may contribute to the pathogenesis of hypospadias and thus requires in-depth research.

## Data Availability Statement

The original contributions presented in the study are included in the article/supplementary material, further inquiries can be directed to the corresponding author/s.

## Ethics Statement

The studies involving human participants were reviewed and approved by Ethical Standards of the Institutional Review Board of Guangzhou Women and Children's Medical Center (NO. 39401). Written informed consent to participate in this study was provided by the participants' legal guardian/next of kin.

## Author Contributions

FD designed experiment. YL, WF, KF, XZ, WJ, NW, GL, and FD collected samples and conducted the study. YZ and XZ analyzed the data. YL and FD wrote the paper. All authors have read and approved the manuscript.

## Funding

FD thanks the fund from Guangzhou Institute of Pediatrics/Guangzhou Women and Children's Medical Center (Grant No. 0190026) and Science and Technology Project of Guangzhou (Grant No. 202102010238).

## Conflict of Interest

The authors declare that the research was conducted in the absence of any commercial or financial relationships that could be construed as a potential conflict of interest.

## Publisher's Note

All claims expressed in this article are solely those of the authors and do not necessarily represent those of their affiliated organizations, or those of the publisher, the editors and the reviewers. Any product that may be evaluated in this article, or claim that may be made by its manufacturer, is not guaranteed or endorsed by the publisher.
